# Theoretical Analysis of Pre-Receptor Image Conditioning in Weakly Electric Fish

**DOI:** 10.1371/journal.pcbi.0010016

**Published:** 2005-07-15

**Authors:** Adriana Migliaro, Angel A Caputi, Ruben Budelli

**Affiliations:** 1 Sección Biomatemática, Instituto de Biología, Facultad de Ciencias, Montevideo, Uruguay; 2 Departamento de Neurociencias Integrativas y Computacionales, Instituto de Investigaciones Biológicas Clemente Estable, Montevideo, Uruguay; University College London, United Kingdom

## Abstract

Electroreceptive fish detect nearby objects by processing the information contained in the pattern of electric currents through the skin. The distribution of local transepidermal voltage or current density on the sensory surface of the fish's skin is the electric image of the surrounding environment. This article reports a model study of the quantitative effect of the conductance of the internal tissues and the skin on electric image generation in *Gnathonemus petersii* (Günther 1862). Using realistic modelling, we calculated the electric image of a metal object on a simulated fish having different combinations of internal tissues and skin conductances. An object perturbs an electric field as if it were a distribution of electric sources. The equivalent distribution of electric sources is referred to as an object's imprimence. The high conductivity of the fish body lowers the load resistance of a given object's imprimence, increasing the electric image. It also funnels the current generated by the electric organ in such a way that the field and the imprimence of objects in the vicinity of the rostral electric fovea are enhanced. Regarding skin conductance, our results show that the actual value is in the optimal range for transcutaneous voltage modulation by nearby objects. This result suggests that “voltage” is the answer to the long-standing question as to whether current or voltage is the effective stimulus for electroreceptors. Our analysis shows that the fish body should be conceived as an object that interacts with nearby objects, conditioning the electric image. The concept of imprimence can be extended to other sensory systems, facilitating the identification of features common to different perceptual systems.

## Synopsis

This paper analyzes the contribution of the body and skin conductance of weakly electric fish in shaping the electric image, using a realistic computational model.

Object recognition, a relevant issue in sensory systems, is not yet fully understood. How pre-receptor mechanisms and interactions between objects shape images is of interest in all sensory systems, leading to general concepts and a specialized jargon. The authors rescue the generality of two concepts for understanding sensory systems. These concepts were introduced early in electroreception research: object perturbed field (change in the basal field generated by the presence of an object), and imprimence (equivalent sources produced at the location of the object). The fish body is an object: generating its imprimence, modifying the basal field, and interacting with other objects. Analogously, the human body interferes, reflecting light or sound in the generation of visual and acoustic images.

The contribution of internal and skin conductivities in image generation has been controversial since the seminal work of Lissmann and Machin in 1958. We show that the high internal conductance of the fish increases and redirects the currents that illuminate objects, increasing and shaping the electric image. Skin resistance mainly influences image magnitude.

## Introduction

Electroreceptive fish detect nearby objects by processing the information contained in the pattern of electric currents through the skin. In weakly electric fish, these currents result from a self-generated field, produced by the electric organ discharge (EOD). Local transepidermal voltage or current density is the effective stimulus for electroreceptors. The distribution of voltage or current on the sensory surface of the fish's skin is the electric image of the surrounding environment [1–3]. From this image, the brain constructs a representation of the external world. Therefore, to understand electrolocation it is necessary to know the image-generation strategy used by electrolocating animals.

Theoretical analysis of image generation has yielded realistic models that predict with acceptable accuracy the electrosensory stimulus [4–12]. One general conclusion of previous reports is that the skin conductance and the conductivity difference between the internal tissues of the fish and the water are the main factors shaping the electric image: the seminal paper by Lissmann and Machin [13] started a long-lasting controversy about the roles of these factors. Lissmann and Machin argued that if “… the fish has approximately the same conductivity as the water and that it does not appreciably distort the perturbing field (i.e., does not produce an image of the image), the potential distribution around the fish due to the perturbing field can be calculated.” However, several reports [3,7,14] have indicated that the internal conductivity of freshwater fish is high with respect to the surrounding water, and that the high conductance of internal tissues is critical for enhancing the local EOD field as well as for generating the centre-surround opposition pattern that characterizes electric images and that is coded by primary afferents [15].

Experimental studies in pulse gymnotids have confirmed theoretical predictions, showing that the high conductivity of the fish body funnels the self-generated current to the perioral region, where an electrosensory fovea has been described on the basis of electroreceptor density, variety, and central representation [16]. This funnelling effect enhances the stimulus at the foveal region. In addition, two different types of skin have been described in some electric fish of the family Mormyridae: the low-conductance mormyromast epithelium where electroreceptors are present, and the high-conductance non-mormyromast epithelium where electroreceptors are absent [7,17]. The mormyromast epithelium is found on the head in front of the gills, as well as along the dorsum of the back and along the ventral surface of the trunk. The non-mormyromast epithelium is found along the sides of the trunk. This heterogeneity of skin conductance introduces another factor shaping physical electric images.

This article describes a realistic modelling study of the effect of the internal and skin conductance on electric image generation in *G. petersii*. We have calculated the electric image of a metal object on a simulated fish having different magnitudes of conductances for internal tissues and skin. While the high conductivity of the fish body enhances the electric image by a combination of mechanisms, the skin conductance appears to optimize the transcutaneous voltage modulation by nearby objects. In contrast, transcutaneous current increases monotonically with skin conductivity. These results suggest that transcutaneous voltage is the critical proximal stimulus for electroreceptors.

We generalize two concepts: “object perturbing field” and “imprimence,” introduced early in electroreception research [13], to other sensory systems. An object perturbs an electric field as if it were adding a new field to the basal one. This perturbing field can be considered as equivalent to a certain distribution of electric sources. This distribution is referred to as an object's imprimence.

## Results

**Figure 1:**
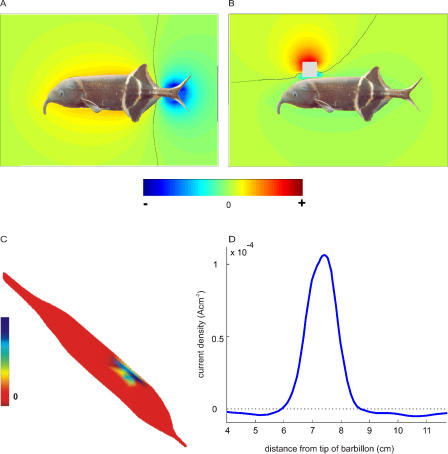
Image Generation in a Fish with Realistic Internal Conductivity and Homogeneous Highly Conductive Skin (A) The coloured background represents the difference in voltage between each point surrounding the fish and an infinitely distant point, using a non-linear arctangent colour scale (used to highlight values close to zero) shown in the colour bar below for the basal field (in the absence of objects). The black line shows the zero equipotential surface, which is perpendicular to the axis of the EO equivalent dipole distribution. (B) A similar coloured representation shows the perturbing field (i.e., the field in the presence of the object minus the basal field) produced by a metal cube (1 cm^3^) close to the skin (0.5 mm). The black line shows the zero equipotential surface, which is perpendicular to the axis of the object equivalent dipole distribution. (C) Electric image of the metal object depicted in a colour map on the modelled realistic fish from a *scorci* view. (D) Electric image along the intersection of the skin with the sagittal plane, illustrating its “Mexican hat” profile.

Electric fields and images generated by metal objects were described in previous reports (reviewed by [3]). In Figure 1, we present results obtained with a realistic fish model and a metal cube, as a reference for the following simulations. Figure 1A shows the basal field, i.e., the field generated by the EOD in the absence of objects. Since all the components of the scene are purely resistive elements, the electric field generated by the EOD has a constant spatial distribution and thus can be described with a static analysis. Therefore, the EOD has been represented by a DC current flowing caudal to rostral along the electric organ (EO). The isopotential lines run closely parallel to the skin, and the distance between them diminishes close to the tip of the “barbillon,” a finger-like extension of the lower lip found in some mormyrid fish. This indicates that field strength and, consequently, current magnitudes are larger at the tip of the barbillon, due to edge effects. The barbillon may be thought of as an “electrosensory fovea” [16,17]. Figure 1B shows the distortion of the basal field (i.e., the object perturbing field) produced by the presence of the cube. This distortion depends on the characteristics of the object and the basal electric field in its neighbourhood. The object perturbing field shown in Figure 1 could also be produced by a set of dipoles oriented almost perpendicular to the fish skin at the point closest to the cube (object imprimence) [13]. The electric image is the difference between the current densities through the skin in the presence and the absence of the object (Figure 1C). Note that the currents increase (positive values) in the skin facing the cube and decrease in a larger surrounding region, producing a “Mexican hat”-type image that can be seen in the graph of Figure 1D, which shows a profile of the image along a line on the sagittal plane. Thus the object image is distributed over a large part of the sensory surface and is not restricted to just the area of skin facing it.

To study the effect of the skin and internal conductances on the generation of the electric image, we departed from the situation proposed by Lissmann and Machin (1958), in which all fish tissues have the same conductivity as the water. Secondly, we studied the effect of changing internal conductivity, while maintaining a skin conductance that was very high and therefore of negligible effect. Thirdly, for an internal conductivity similar to that experimentally determined, we studied the effect of changing skin conductance as if it were uniform along the fish surface. Finally, we compared results obtained with homogeneous skin conductances and those obtained with the heterogeneous distribution of the skin conductances that is present in *G. petersii.*

### Images as a Function of Fish Internal Conductivity

We have proposed that the low resistivity of the fish body is a very important factor for the shaping of the electric image. To assess its contribution, we simulated electric images for fish having a high skin conductance but with different internal conductivities.

**Figure 2:**
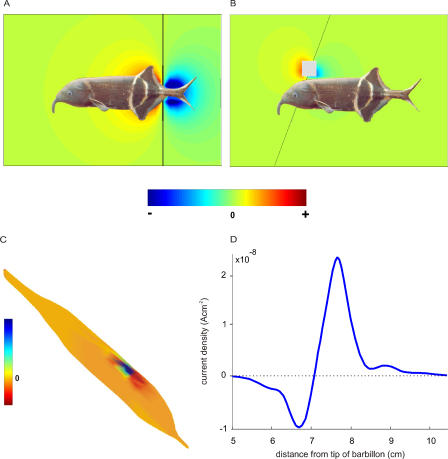
Image Generation in a Fish with Internal Conductivity like That of Water and with a Homogeneous Highly Conductive Skin The black bars show the zero equipotential surfaces as in Figure 1. (A) Basal field (in the absence of objects). (B) Perturbing field produced by the same scene as in Figure 1B. (C) Electric image of the metal object depicted in a colour map on the modelled transparent fish from a *scorci* view. (D) Electric image along the intersection of the skin with the sagittal plane.

We first modelled a fish with an internal conductivity equal to that of the water, as assumed by Lissmann and Machin [13]. This is described as a “transparent fish.” In this case, the conductivity of the surrounding medium is homogeneous except for the object. Thus, the images calculated as the distribution of the current density across a virtual sensory epithelium are the perturbing fields at the surface of the skin. The basal field generated by the EO is similar to the field of a dipole in a homogeneous medium (Figure 2A). Consequently, and in contrast to the real situation, the isopotentials lines do not run closely parallel to the skin, and the field at the tip of the barbillon does not show an edge effect. Figure 2B shows the perturbing field (the field in the presence of the object minus the basal field) produced by a metal cube close to the skin. The imprimence of the object is equivalent to a certain distribution of dipoles located at the object site, no longer oriented perpendicular to the skin, in contrast to the naturally realistic case (see Figure 1B).

**Figure 3:**
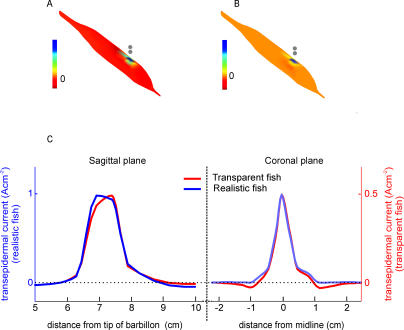
The Effect of Internal Conductivity on the Image Generation of a Dipole (A) Electric image of a dipole placed at 0.5 mm from a “transparent” fish seen from a *scorci* view; the modelled dipole axis is perpendicular to the longitudinal axis of the fish. (B) Same scene as (A) for fish with realistic internal conductivity. (C) Electric image (transcutaneous current density) along the intersection of the skin with the sagittal plane (left), and the coronal plane (right), for the same dipole as in (A) and (B). Red traces show the images on a transparent fish, while blue traces correspond to a fish with realistic internal conductivity. Note that the ordinate for the realistic fish (left) is twice that for the transparent fish (right).

Comparison of Figures 1 and 2 shows that the direction of the field is nearly parallel to the transparent fish body, and nearly perpendicular to the real fish body. This indicates that the conductivity of the fish body distorts the field produced by the EO. It is worth noting that the internal conductivity of the fish not only funnels the current rostrally but also exerts an effect on the field direction generated at the object location: as a consequence, the electric image is more symmetric. Comparison of image profiles along a sagittal plane (Figures 1D and 2D) shows an enhancement of the image amplitude produced by the presence of the fish body. The body exerts this effect in two ways: a) by increasing the local field in the vicinity of the object, therefore increasing the perturbing field and its imprimence, and b) by introducing an impedance gradient at the site of the sensory surface. Previous research has shown that the amplitude of the image generated by a dipole increases up to two times when the fish/water conductivity ratio increases [18]. To test this mechanism, we calculated the image of a dipole perpendicular to the skin of a transparent fish (Figure 3A, with the negative pole facing the skin), comparing it with the image of the same dipole on a fish with normal internal impedance (Figure 3B). While the waveform remains similar as shown in the current profiles along the skin intersecting with the sagittal and coronal planes, the amplitude of the profile for the realistic fish is twice that for the transparent fish (Figure 3C).

**Figure 4:**
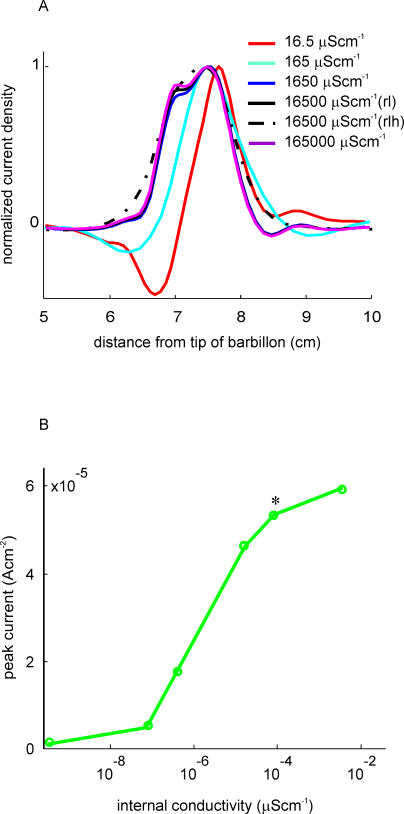
The Effect of Internal Conductivity on Electric Image Generation (A) Normalized electric images of the same metal cube (identical position) on fish with different internal conductivities. Red: 16.5 μScm^−1^ (the same as water conductivity), cyan: 165 μScm^−1^, blue: 1,650 μScm^−1^, black: 16,500 μScm^−1^ (normal conductivity), magenta: 165,000 μScm^−1^. The skin is modelled for all cases, with a homogeneous conductivity of 500,000 μScm^−1^. The dashed line shows the case of a fish with realistic internal conductivity and skin conductivity distribution. rl, realistic internal conductivity; rlh, realistic internal conductivity, heterogeneous skin distribution. (B) Peak amplitude of the electric image of a metal cube (1 cm^3^) placed at 0.5 mm from the fish, as a function of body internal conductivity. The difference in the peak amplitude of the electric image corresponding to the realistic internal conductivity fish shown in this figure and that shown in Figure 1 is due to the use of two compartment bodies (see Materials and Methods).

Figure 4A shows the normalized electric image of a metal cube calculated for fish with different body conductivities. In order to maintain a constant electric source, the tail region was modelled as an independent compartment with realistic internal conductivity. As shown in the normalized images (Figure 4A), both edges shift rostrally with a predominant shift of the rostral border, so that the image becomes wider as body conductivity increases. In addition, the shape of the profile, which initially consists of two main deflections (one caudal positive and one rostral negative), becomes more symmetric, resembling a Mexican hat. The amplitude of the image is an increasing function of body conductance (Figure 4B). These changes are correlated with an increase in the magnitude and a change in the direction of the basal field around the object.

### The Effect of Skin Conductance

**Figure 5:**
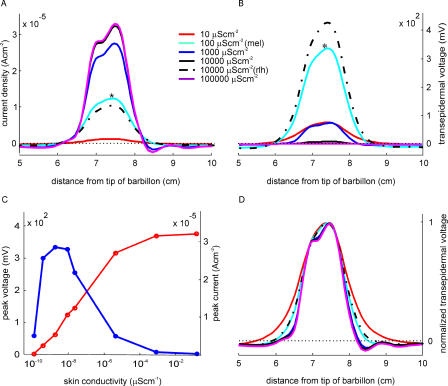
The Effect of Skin Conductivity on Electric Image Generation (A) Transcutaneous current density (electric image) of a metal cube (1cm^3^) placed at 0.5 mm from the skin, modelled on skin with different conductivities. Red: 10 μScm^−2^, cyan: 100 μScm^−2^ (similar to mormyromast epithelium), blue: 1,000 μScm^−2^, black: 10,000 μScm^−2^, magenta: 100,000 μScm^−2^. All these fish have an internal conductivity of 3,300 μScm^−1^. Dashed line shows the case of a fish with realistic internal conductivity and skin conductivity distribution. (B) Transcutaneous voltage calculated from the transcutaneous current densities shown in (A), using the same colour code. (C) Current peak (right axis, red trace) and voltage peak (left axis, blue trace) as a function of skin conductivity for fish with homogeneous skin. (D) Normalized plot for (B), using same colour code. mel, mormyromast epithelium-like conductivity; rlh, realistic internal conductivity, heterogeneous skin distribution.

To assess the contribution of the skin to image formation, we studied the effect of different uniform skin conductances for a fish with normal internal conductivity. For very low skin conductivity, the transepithelial currents produced by the EO are negligible (Figure 5): the current short-circuits inside the fish because it cannot flow through the skin. The transepithelial current increases with the skin conductivity, approaching an asymptotic value (red trace in Figure 5A and 5C). Since transcutaneous voltage is the quotient of the current density divided by skin conductance, voltage increases differently with skin conductance, rising to a maximum and then decreasing (blue trace in Figure 5B and 5C). The value of skin conductance at which voltage reaches a maximum, 100 μScm^−2^, is close to the actual measured value for the mormyromast epithelium. This suggests that electroreceptors operate in a voltage detection mode rather than in a current detection mode. The normalized curves in Figure 5D show that the image is smoother and wider as the skin conductance decreases. Continuous traces correspond to uniform skin conductances, where the cyan one is the closest to the mormyromast epithelium value. Realistic electric images were calculated as a reference, using the distribution of conductivities determined experimentally (dotted traces) [7].

## Discussion

Animals extract information from the environment and from their own bodies by analyzing changes in the patterns of energy impinging on their sensory surfaces. In that sense, it can be affirmed that to see is to reconstruct visual scenes from a light pattern on the retina or to hear is to extract auditory scenes from sound patterns at the cochlea [19]. Similarly, electric sensing is to reconstruct electric scenes from the pattern of electric currents through the skin.

In electrosensory perception, each object generates a signal that results from the deformation that its presence causes in an electric field. This deformation is a virtual field, called “object perturbing field” by Lissmann and Machin [13]. The object perturbing field is not directly measurable, but computable as the electric field in the presence of the object minus the electric field in its absence, also called “basal field.” As any electric field, the object perturbing field can be considered as caused by an electric source, which is equivalent to the presence of the object.

The “imprimence” of an object, an expression also coined by Lissmann and Machin [13] referring to the electric sources equivalent to the object, not only generates an image but also a change in the field that interacts with other objects. Thus, the effect of a given object not only generates its own image but also modifies the images of other objects [10]. There are theoretical and experimental reasons indicating that the fish body is also an object, and that this is of particular importance since it is an object that is always present as a major determinant of sensory imaging [5,7,18,20]. This leads to the proposition that the fish body, by its presence and movements, constitutes a critical pre-receptor mechanism that conditions sensory signals [16,21]. We, therefore, discuss here the effect of relevant components of the fish's body on image generation.

### The Effect of the Fish's Internal Conductivity

**Figure 6:**
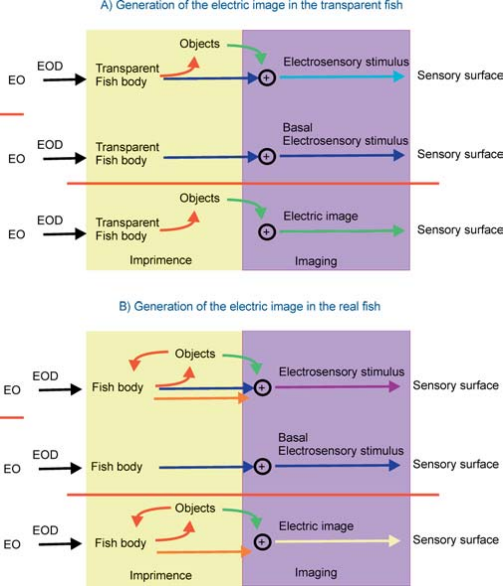
Schematic Representation of Electric Image Generation First row, generation of stimulation in the presence of the object; second row, basal stimulation in the absence of objects; third row, sensory image. (A) Fish with water-like internal conductivity. Imprimence generation (yellow boxes) precedes image generation (purple boxes). A field perturbation (green arrows) is induced as a consequence of the object interaction with the basal field (dark-blue arrows). The electric image is the difference between the perturbing (light-blue arrow) and the basal fields at the skin. (B) Fish with realistic internal conductivity. The interaction of the body with the field perturbed by the object (red arrows) introduces another component (orange arrow) to the electrosensory stimulus (magenta arrow). The electric image (yellow arrow) is the electrosensory stimulus minus the basal field (blue arrow, representing the sum of the effects of the fish body and the object in the presence of each other). (See Discussion for explanation.)

The imaging process consists of two steps: imprimence generation (yellow boxes in Figure 6) and image generation (purple boxes in Figure 6). The simplest example occurs in a “transparent” fish, isoconductive with water. The electric image (green arrow in Figure 6A) is the difference between the electrosensory stimulus generated in the presence of an object (light-blue arrow in Figure 6A) and the electrosensory stimulus generated in the absence of that object (dark-blue arrow in Figure 6A). The latter is referred to as the basal stimulus because it is caused by the basal field. Since, in the case of the transparent fish, the basal field is not distorted by the fish body, the electric image results from the projection on the skin of a field perturbation induced only as a consequence of interaction of the object with the basal field (green arrows in Figure 6A). Then, in a transparent fish, image formation can be described as a simple process consisting of two steps: a) the generation of a field by the EO and b) the deformation of this field by the presence of the object.

However, in nature, the basal field is different from that produced by the EO in a homogeneous medium, because it is affected by the inextricable presence of the fish body. Similarly, the object perturbing field is also affected by the fish's body. This interaction (red arrows) produces two extra components that add to the basal electrosensory stimulus (dark-blue arrow): the perturbing field of the object (green arrow) and the perturbing field of the fish body (orange arrow). This resulting field acting on the skin is the electrosensory stimulus (magenta arrow). To calculate the electric image, we subtracted the effect of the basal stimulus (fish body alone, blue arrow). Thus, the electric image (yellow arrow) results from the addition of the perturbing field of the fish's body in the presence of the object (orange arrow) plus the perturbing field of the object in the presence of the fish's body (green arrow). When the object is large enough and surrounds the fish, its effect becomes very important, having a strong influence on the overall pattern of current flow. This is the case when the fish chooses to stay in confined spaces that are frequently its preference in the natural habitat, or in the tube-shaped shelters commonly used in captivity. The fish's positioning of its body in this manner strongly affects the electric images of objects and electrosensory responses [22].

When the object is relatively small or far from the fish body, the loop between the object and the fish body opens, because the influence of the field of the object on the fish body becomes negligible compared to the basal field. Consequently, the scheme of image generation is the same as in the case of the transparent fish. However, the basal field illuminating the object is different than that in the case of a transparent fish and so is the image.

### The Effect of Skin Conductivity

The skin conductance is the other important factor shaping the electric image. A homogeneous decrease of the skin conductance causes: a) a decrease of the transepithelial current density, b) an increase of the transepithelial voltage up to a maximum at the range of natural skin conductivity, c) a decrease of the relative slope of the flanks of the image, and d) an increase of the centre region of the “Mexican hat” profile.

For measuring electrosensory stimulus, either local field (equivalent to current flow) or transcutaneous voltage has often been used indiscriminately [1,20,23]. However, our results indicate that current density and voltage are not equivalent stimuli. The transepithelial change in voltage caused by an object is the maximum within the range of skin conductances that are actually measured in the mormyromast epithelium of *G. petersii* (70–500 μScm^−2^ [7]), suggesting that the low conductance observed in the mormyromast epithelium might be an adaptation for optimizing voltage sensing. This low conductance of the mormyromast epithelium is caused by a thin layer of tightly packed epithelial cells [24], which makes the mormyromast epithelium up to ten times more resistant than the non-mormyromast epithelium [7]. If receptors electrically shunt their low conductance non-sensitive surroundings, transcutaneous voltage could be considered to be the meaningful parameter of the stimulus. Experimental measurements testing this hypothesis should be done.

Our study also shows a consistent decrease in the relative slope of the flanks of the image and an increase in the centre region of the “Mexican hat” profile with increasing skin resistance (see Figure 5D). In this study, restricted to single conductive objects close to the skin, these changes are rather small, indicating that the main factor for determining object image shape is the internal conductance of the fish body.

### The Generality of the Concepts of Object Perturbing Field and Imprimence

Object recognition is an important issue in all sensory systems (including electrical perception), but it is not well understood. The comparative study of different sensory systems leads to general concepts and a language that could potentially be shared by researchers in different systems. In this paper, we focus on peripheral imaging mechanisms, a subject common to sensory systems. We focus in particular on the way in which pre-receptor mechanisms and interactions between different objects in a given scene shape the image.

We emphasize two concepts that were introduced early on in electroreception research [13]: object perturbing field and imprimence. An object perturbs an electric field as if it were a distribution of electric sources. The equivalent distribution of electric sources is referred to as an object's imprimence. Object perturbing field is a concept that relates to reflections and refractances in vision, echoes in audition, etc. In the same way that objects of different impedance than the water modify an electric field, objects in a visual scene modify the illumination. Similarly, echoes and resonances produced by objects modify the distribution of sound in an auditory scene. For example, the sound of a pulsed string on a guitar is greatly modified by the resonance of the box, giving the sound a characteristic timbre. The concept of imprimence can be extended in the same way. Objects producing reflexions, refractances, echoes, and resonances can be considered new sources of energy.

The imprimence produced by the animal's own body acts as a pre-receptor mechanism. The fish body can be considered as an object that interacts with other objects in the scene, generating an imprimence that through the perturbing field modifies the basal field of the scene and, consequently, the imprimence of the other objects. Many species of fish (including Mormyrids) hear underwater due to the imprimence produced by air-filled sacs such as the swimbladder [25]. In a less fundamental way, our body changes the visual image by interfering with and reflecting light, modifying the images of nearby objects. In addition, the interactions between objects and the perturbations of the fields by the imprimences of other objects are used to extract information from a scene. For example, the imprimence of the external ear modifies the incoming sound, allowing for the computation of the altitude of a source [26].

Animal senses explore nature using a limited number of types of energy and receptors with limited dynamic ranges. This constrains and conditions the representation of external reality according to the capabilities of each animal. Humans circumvent these limitations by creating artificial systems, such as radar or sonar, which expand the repertoire of representable qualities of objects. The concepts of imprimence and perturbing field may be applied to the design of artificial sensory systems. It is a common practice to deal with interactions between objects and the perturbations of the fields by the imprimences of other objects as undesirable interference. Nevertheless, evolution has developed neural operations that use images resulting from object interferences as a source of information, in some cases using this to infer object attributes. In these cases, interference between objects may increase the amount of available information contained in the image. Development of the theory of peripheral imaging is a necessary step for the design of computational procedures, allowing the extraction of a larger amount of information from the same signals.

### Conclusions

The electric image of an object results from the projection on the skin of a virtual field caused by the presence of an object, in a given electrosensory scene.

The fish's large internal conductance (compared with water) causes a rostral funnelling of the current. This leads to an increase in the imprimence of objects close to the rostral regions of the fish and, consequently, to an increase in the amplitude of their images.

The large difference in conductivity between the inside and outside of the fish forces the field to be almost perpendicular to the sensory surface and, consequently, makes the shape of the image more symmetrical.

An object modifies the field of other objects immersed in the same global field. The fish body itself is a major object, inherent to the process of image generation. Thus, a global field results from the reciprocal interaction between the fish body and nearby objects.

The conductance of the skin changes the shape of the image only slightly, but drives the amplitude (considered as the distribution of transepithelial voltages) close to its maximum, for a given set of other electrical parameters. This result suggests that the high resistance of the mormyromast surface, a property conferred by a thin layer of tightly packed epithelial cells, may serve to optimize object images.

The use of a realistic computational model has allowed us to settle the controversy about the relative importance of the internal and skin conductivities in the determination of the magnitude and shape of the electric image, an issue that has been debated since the seminal paper by Lissmann and Machin [13].

We propose that the concepts of perturbing field and imprimence [13] may be usefully applied to the analysis of other sensory systems and the design of artificial ones.

## Materials and Methods

### The model.

Simulations were run using a program written to simulate the electric image in weakly electric fish (i.e., the currents through the fish skin), which uses the Boundary Element Method (BEM [27], as proposed by Assad [4], and has been described previously [10,28]). This program allows the determination of the electric field and the electrosensory image in a given environment (scene), calculating the currents through the skin. A scene may include objects (other than the fish) of different conductivity, shape, and size, and is defined by setting the geometry and location of one or more electric fish and objects. Water, internal, and skin conductivities can be specified as required. When the skin conductivity is not homogeneous, different regions can be defined using a graphic interface. Complex shapes, including the fish body, are approximated by a surface composed by triangles. Although the fish shape is kept constant throughout this article, the model allows its modification if required. Once the scene is determined, the potentials and current densities through the skin of the fish and through the objects are calculated. The graphic presentations were made by Matlab standard subroutines.

### Changes in internal conductivity and skin conductance.

We studied the effect of the skin and internal conductivity on the electric image in the presence of a metallic (high conductance) cube placed symmetrically to the sagittal plane and facing the dorsal skin 0.5 mm away. Water conductivity was kept at 16.5 μScm^−1^.

To assess the influence of the internal conductivity of the fish body, different values ranging from that equal to surrounding water conductivity (in which case the fish may be considered transparent) to 16,500 μScm^−1^ were examined, including the value experimentally determined (3,300 μScm^−1^). In order to maintain a constant electric source, tail and body regions were modelled as independent compartments, maintaining the tail with a realistic internal conductivity while applying different values for the body. In these cases, the conductance across the model skin was set low enough to be considered irrelevant.

To study the influence of the skin conductance, we explored the effect of different skins with homogeneously distributed conductances ranging from 10–100,000 μScm^−2^ and a natural-like skin with heterogeneous conductance distribution. The internal conductivity in this case was close to that experimentally determined (3,300 μScm^−1^).

Two singular conditions were used for comparison purposes: a) when the fish model has experimentally determined conductances (where the fish body exerts its normal effect on the electric image); and b) when it has water-like conductances (i.e., where the fish body exerts no effect on the electric image).

## Abbreviations

EO: electric organ
EOD: electric organ discharge
